# Supplementary material for: Nontargeted Screening for Flavonoids in Salicornia Plant by Reversed-Phase Liquid Chromatography–Electrospray Orbitrap Data-Dependent MS2/MS3
Source: Molecules. 2023 Mar 28;28(7):3022. doi: 10.3390/molecules28073022 (PMC10096158; doi:10.3390/molecules28073022)
Supplement: Supplementary file 1 [file molecules-28-03022-s001.zip › molecules-2257533-supplementary.pdf]

Supplementary Materials.

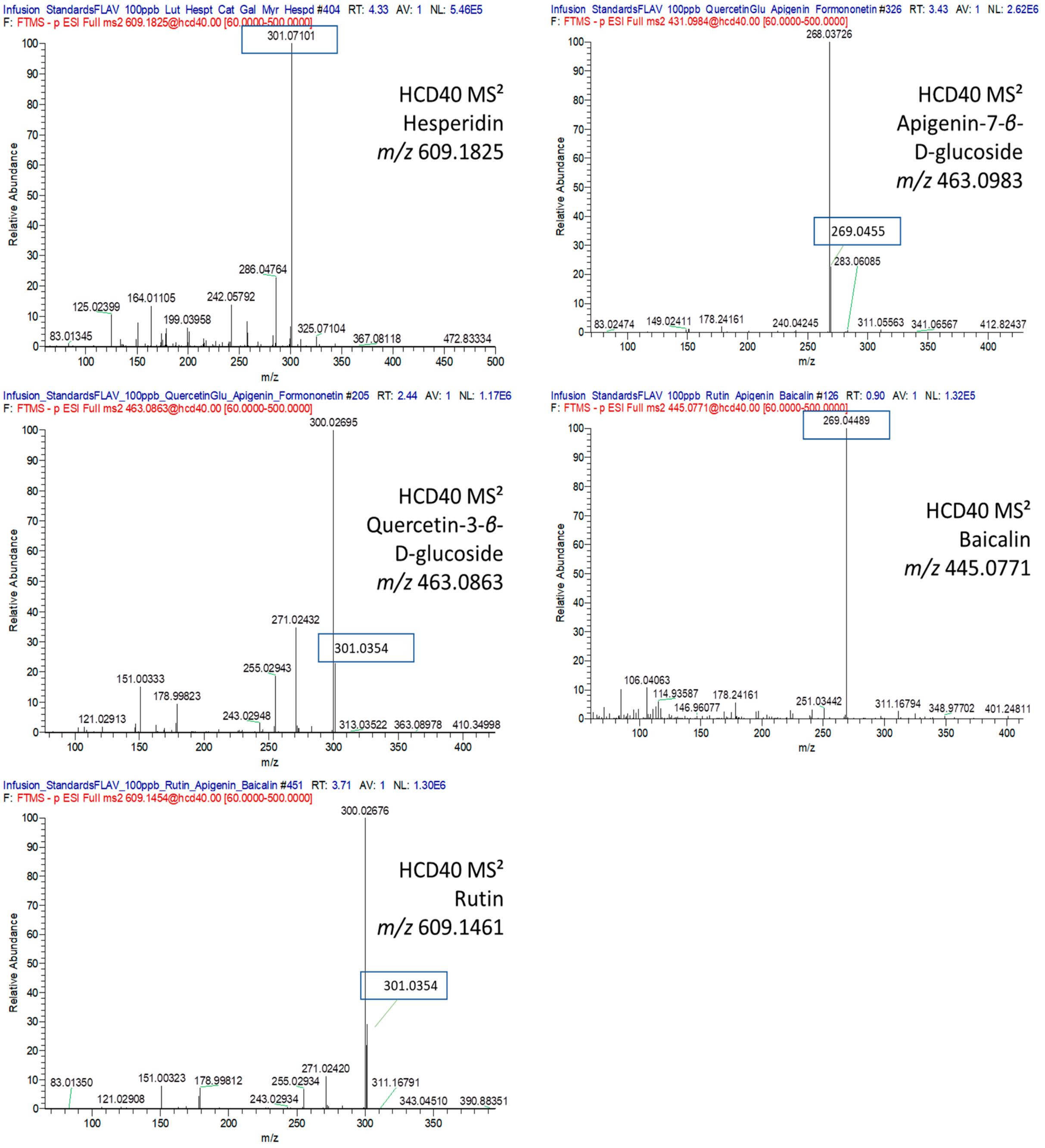

Figure S1. HCD40 and CID30 MS² spectra acquired after infusion of five glycosylated flavonoids, namely hesperidin, apigenin-7-*β*-D-glucoside, rutin, quercetin-3-*β*-D-glucoside and baicalin.

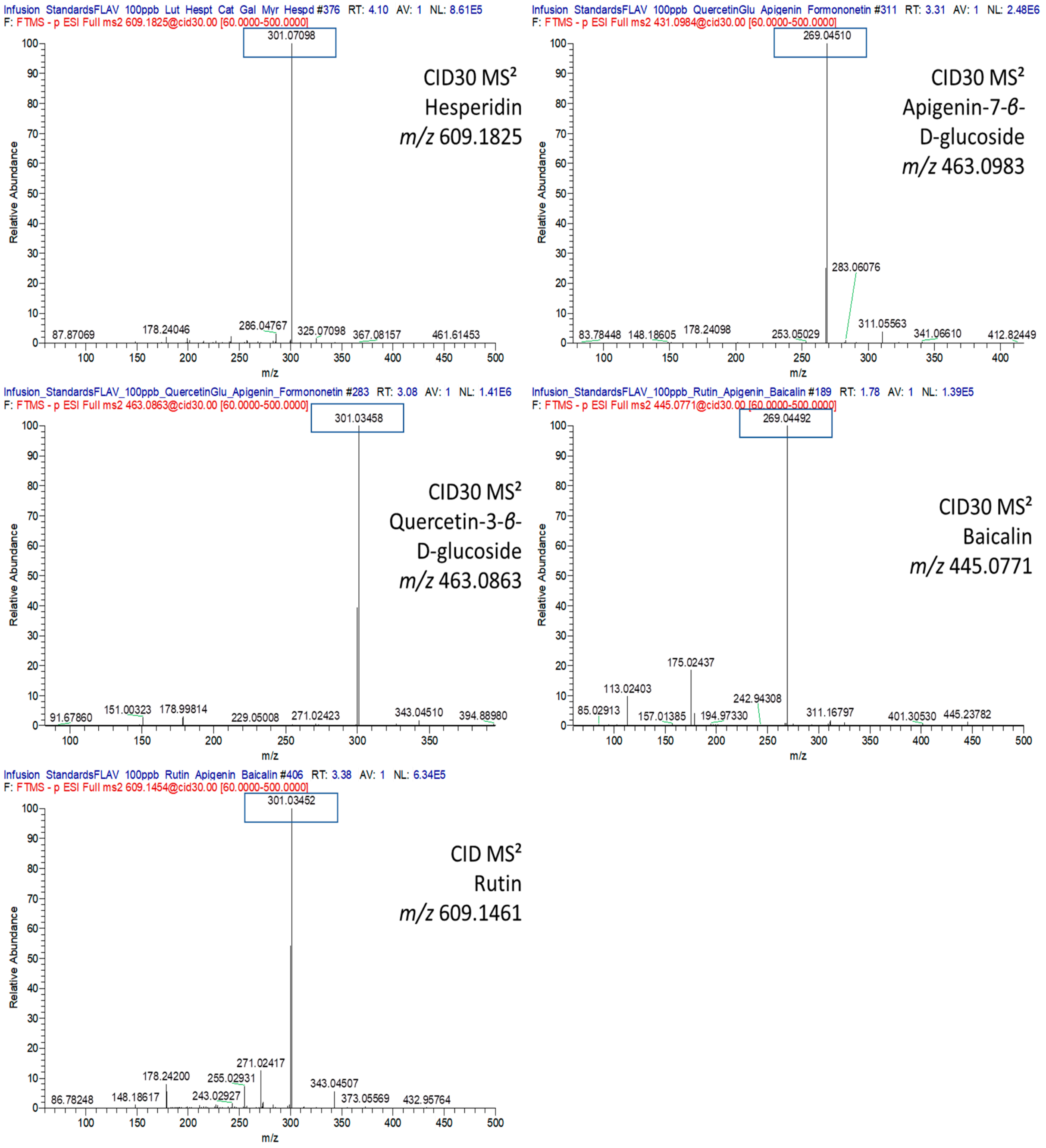

Figure S1. *Cont.*

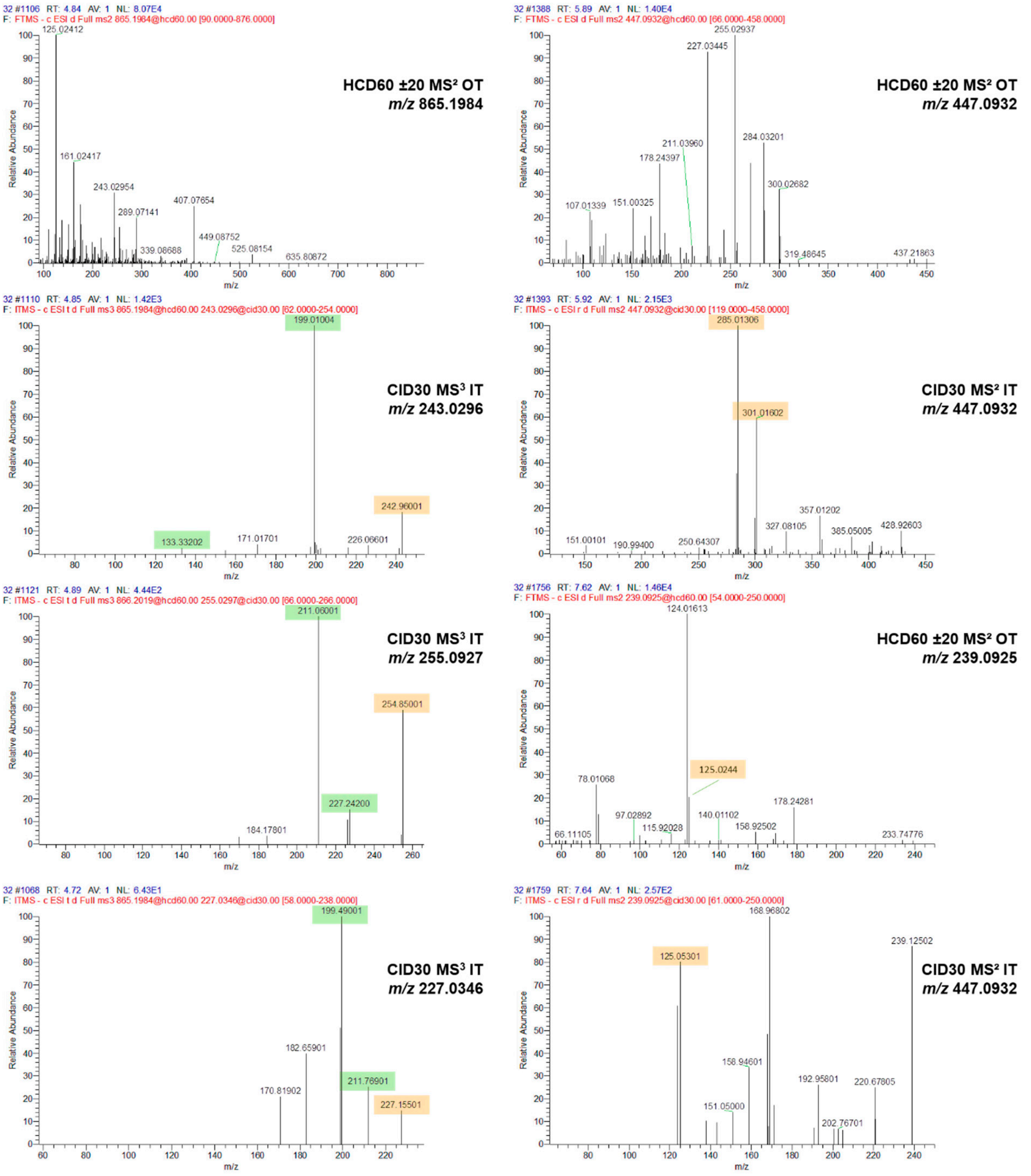

Figure S2. Fragmentation data acquired in HCD60 ±20 MS^2 orbitrap (OT) scans and CID30 MS^2/MS^3 ion-trap (IT) scans after applying the non-targeted Top3 ddMS^2/MS^3 method. Candidate-flavonoids lower than *m/z* 600 were fragmented in CID30 MS^2 IT scans whereas CID MS^3 scans were triggered for bigger compounds showing characteristic fragment ions in their prior HCD60 ±20 MS^2 scans. CID30 MS^3 scans were triggered on the biggest characteristic fragment ions detected in HCD60 ±20 MS^2 scans whereas CID30 MS^2 scans were applied on the precursor ions for detected flavonoids lower than *m/z* 600.

Table S1. Candidate-flavonoids detected in the model grape seed sample by the use of the non-targeted ddMS²/MS³ method.

Flavonoids	Formula	Monoisotopic mass (Da)	mass error (ppm)	$[M-H]^-$ *m/z*	10 mM NH_4Ac pH 5.4	RT (min)	Nb of CFI (/15)
Gentisic acid*	C7 H6 O4	154.02655	-0.39	153.0193	3.76E+07	1.958	4
4-hydroxymandelic acid*	C8 H8 O4	168.04223	-0.14	167.0345	1.47E+07	1.679	3
**Gallic acid**[1]	C7 H6 O5	170.02144	-0.51	169.0142	2.37E+08	1.306	6
Vanillyl mandelic acid*	C9 H10 O5	198.05282	0	197.0455	5.40E+07	5.305	5
3,4,5-trimethoxyhydrocinnamic acid*	C12 H16 O5	240.09979	0.07	239.0925	5.62E+05	7.592	4
**Epicatechin**[1]	C15 H14 O6	290.07905	0.05	289.0720	5.19E+07	4.555	3
**Catechin**[1]	C15 H14 O6	290.07905	0.05	289.0720	3.90E+07	2.981	3
**Quercetin**[1]	C15 H10 O7	302.04268	0.08	301.0354	1.75E+06	6.746	11
**Gallic-acid-hexoside**[1]	C13 H16 O10	332.07438	0.12	331.0671	6.12E+07	1.359	4
**Syringic acid hexoside**[1]	C15 H20 O10	360.1057	0.14	359.0984	1.23E+07	4.506	2
Unknown	C20 H24 O6	360.15733	0.12	359.1501	1.85E+06	4.749	2
Unknown	C21 H32 O10	444.1996	0.12	443.1923	3.45E+07	2.476	9
**Quercetin-rhamnoside**[1]	C21 H20 O11	448.10048	-0.19	447.0932	2.01E+06	5.807	3
**Eriodictyol-hexoside**[1]	C21 H22 O11	450.11619	-0.04	449.1089	1.13E+07	4.703	4
**Catechin-hexoside**[1]	C21 H24 O11	452.13188	0.04	451.1246	6.72E+06	2.922	3
**Quercetin-*3-β-D*-glucoside**[1]	C21 H20 O12	464.09544	-0.07	463.0863	6.43E+06	5.519	10
**Isorhamnetin-hexoside**[1]	C22 H22 O12	478.11101	-0.25	477.1037	9.51E+05	5.869	2
Unknown	C26 H36 O11	524.22572	-0.08	523.2184	2.40E+07	5.643	7
**Type B procyanidin**[1]	C30 H26 O12	578.14247	0.08	577.1353	3.42E+07	3.908	10
**Type B procyanidin**[1]	C30 H26 O12	578.14247	0.08	577.1353	2.43E+07	2.629	5

Table S1. *Cont.*

Flavonoids	Formula	Monoisotopic mass (Da)	mass error (ppm)	[M-H]$^-$ *m/z*	10 mM NH_4Ac pH 5.4	RT (min)	Nb of CFI (/15)
**Type B procyanidin[1]**	C30 H26 O12	578.14247	0.08	577.1353	2.21E+07	3.58	5
**Type B procyanidin[1]**	C30 H26 O12	578.14247	0.08	577.1353	1.90E+07	2.225	5
**Type B procyanidin[1]**	C30 H26 O12	578.14247	0.08	577.1353	3.87E+06	5.045	2
Catechin-di-hexoside*	C27 H34 O16	614.1844	-0.46	613.1771	2.59E+06	1.752	2
Catechin-di-hexoside*	C27 H34 O16	614.1844	-1.88	613.1771	9.28E+05	1.558	2
Unknown	C37 H30 O16	730.15346	0.1	729.1461	1.23E+07	5.033	10
Unknown	C37 H30 O16	730.15346	0.1	729.1461	4.95E+06	4.73	10
Unknown	C45 H38 O18	866.20576	-0.06	865.1985	1.15E+07	4.845	11
Unknown	C45 H38 O18	866.20576	-0.06	865.1985	7.63E+06	2.925	8
Unknown	C45 H38 O18	866.20576	-0.06	865.1985	6.47E+06	1.328	8
Unknown	C45 H38 O18	866.20576	-0.06	865.1985	4.03E+06	5.372	10
Cinnamtannin*	C60 H50 O24	1154.26814	-0.92	1153.2610	2.89E+06	4.978	3
Cinnamtannin*	C60 H50 O24	1154.26814	-0.92	1153.2610	2.01E+06	2.245	4

Flavonoids[1] detected in targeted HCD30 MS^2 Analysis. Flavonoids* detected with multiples matches with online and local databases.
